# Campanile Near-Field Probes Fabricated by Nanoimprint Lithography on the Facet of an Optical Fiber

**DOI:** 10.1038/s41598-017-01871-5

**Published:** 2017-05-10

**Authors:** Giuseppe Calafiore, Alexander Koshelev, Thomas P. Darlington, Nicholas J. Borys, Mauro Melli, Aleksandr Polyakov, Giuseppe Cantarella, Frances I. Allen, Paul Lum, Ed Wong, Simone Sassolini, Alexander Weber-Bargioni, P. James Schuck, Stefano Cabrini, Keiko Munechika

**Affiliations:** 1 aBeam Technologies, Hayward, CA 94541 USA; 2 The Molecular Foundry, Lawrence Berkeley National Laboratory, One Cyclotron Road, Berkeley, CA 94720 USA; 3 Biomolecular Nanotechnology Center/QB3, Stanley Hall, University of California, Berkeley, CA 94720 USA; 4 Department of Materials Science and Engineering, University of California, Berkeley, CA 946720 USA

## Abstract

One of the major challenges to the widespread adoption of plasmonic and nano-optical devices in real-life applications is the difficulty to mass-fabricate nano-optical antennas in parallel and reproducible fashion, and the capability to precisely place nanoantennas into devices with nanometer-scale precision. In this study, we present a solution to this challenge using the state-of-the-art ultraviolet nanoimprint lithography (UV-NIL) to fabricate functional optical transformers onto the core of an optical fiber in a single step, mimicking the ‘campanile’ near-field probes. Imprinted probes were fabricated using a custom-built imprinter tool with co-axial alignment capability with sub <100 nm position accuracy, followed by a metallization step. Scanning electron micrographs confirm high imprint fidelity and precision with a thin residual layer to facilitate efficient optical coupling between the fiber and the imprinted optical transformer. The imprinted optical transformer probe was used in an actual NSOM measurement performing hyperspectral photoluminescence mapping of standard fluorescent beads. The calibration scans confirmed that imprinted probes enable sub-diffraction limited imaging with a spatial resolution consistent with the gap size. This novel nano-fabrication approach promises a low-cost, high-throughput, and reproducible manufacturing of advanced nano-optical devices.

## Introduction

The development of plasmonics and nano-optics has recently had a significant impact on research, since it couples light from the far-field to a sub-diffraction limited spot, accessing a so far unreachable parameter space for imaging, spectroscopy and sensing^1–3^. In particular, optical imaging and spectroscopy (Raman, photoluminescence, time-resolved spectroscopy) have benefitted from the development of plasmonics, achieving 20 nm spatial resolution combined with enough signal to noise ratio for single molecule detection and have shown an enormous potential for ultra high sensitivity sensing^4–7^. While the state of the art nanofabrication techniques have made the proof of principle of sophisticated nanoantenna possible^4, 5, 8–16^, the bottleneck for implementing them into actual devices or making nanoantenna-based characterization tools widely available, is the ability to mass fabricate nano-optical antennas in parallel, reproducibly with sub 100 nm resolution, and to place them with nanometer precision into a device. Here, we present a solution to this challenge using state of the art ultraviolet nanoimprint lithography (UV-NIL), demonstrated on currently one of the most advanced optical transformers, the “campanile”, imprinted at the end of a glass fiber tip. We demonstrate the capability to imprint 3D structures with sub-70 nm scale features and sub-100 nm positioning precision, creating functional ‘campanile’ near-field probes.

**Figure 1:**
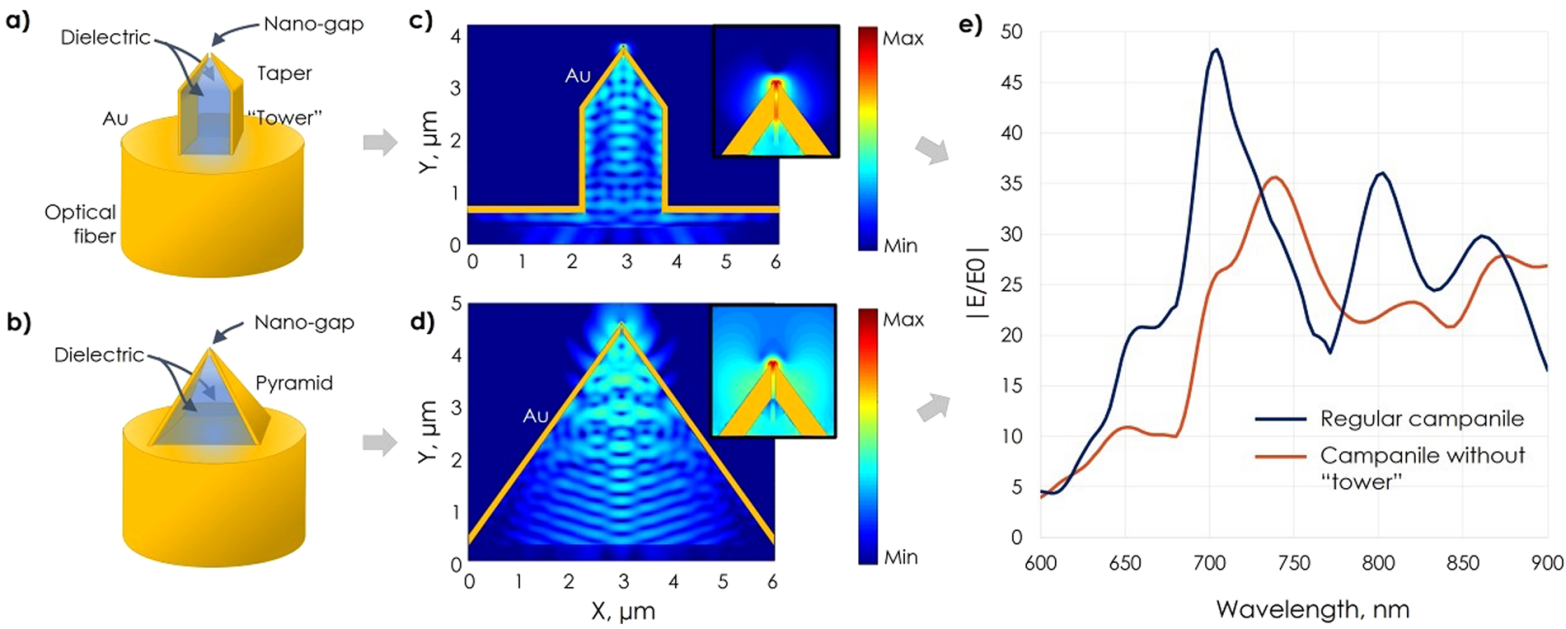
Campanile probe geometry and simulations. Geometries of the regular (**a**) and “tower-less” campanile probe (**b**). The main difference is that the latter lacks a columnar tower between the pyramid structure and optical fiber and has a larger base. FDTD simulations of the electric field amplitude inside the campanile for a regular (**c**) and “tower-less” (**d**) campanile. The insets show close-up views of the hot-spot on the apex. The horizontal extent of the inset is 600 nm, and the width of the campanile gap is 20 nm. Comparison of field enhancements as a function of wavelength for both configurations (**e**).

Optical transformer based near-field probes, also called the “campanile”, are based on the optical transformer concept^17^ that addressed most of the shortcomings of the different near-field optical probe architectures; a strong local electromagnetic field enhancement, efficient far-field to near-field coupling, nanoscale spatial resolution, background-free operation, broadband photon-plasmon coupling and access to polarization properties^18, 19^. The campanile probe is comprised of a three-dimensional (3D) pyramidal metal-insulator-metal (MIM) geometry as shown in Fig. 1a. The tips can support a photon-plasmon conversion efficiency of up to 70% over a broadband wavelength range and have demonstrated sub-40 nm resolution with a normalized intensity enhancement of up to 500 times in the near infrared^20^. Typically, the imaging resolution is dictated by the size of the nano-gap located at the apex of the probe, and the MIM design allows for a bi-directional coupling of light. The campanile probes can be conveniently adopted for hyperspectral imaging and have already enabled nanoscale mapping of photoluminescence heterogeneity in InP nanowires, MoS_2_ monolayers, as well as the sub-wavelength visualization of electromagnetic fields in photonic crystals^21–23^. However, the fabrication of this three-dimensional nano-antennae has remained very complicated and time-consuming, requiring multiple steps of focused ion beam (FIB) milling on the tip of a tapered optical fiber. This severely limits throughput and reproducibility, and restricts the use of campanile tips to only select, priority applications in a research environment.

We have developed a drastically simplified process to fabricate a campanile-like near-field probe at the end of an optical fiber using NIL^24, 25^. NIL is an ideal contemporary fabrication technique for high throughput manufacturing of low-cost campanile probes. Complex photonic and plasmonic devices with high resolution have been successfully fabricated at the wafer level and shown performance comparable to those of similar devices fabricated by electron beam lithography (EBL)^26, 27^. While we have previously demonstrated complex 3D diffractive optical elements on the facet of a fiber by NIL^28, 29^, imprinting of an optical transformer on the cleaved end of an optical fiber poses major technological challenges. First, the imprint must be able to faithfully replicate micrometer scale patterns (the pyramid) and sub-100 nm size features (the gap at the apex) simultaneously. Secondly, the accuracy of co-axial alignment between the campanile pyramid structure and the mode of the single mode optical fiber has to be better than 500 nm. Finally, any residual layers between the optical fiber and the imprinted pyramid structure need to be minimized to thicknesses that are smaller than the wavelength of light to avoid significant losses in performance due to inefficient coupling and forward scattering. We have overcome each of the challenges and developed the process to imprint an optical transformer in a single step, which has eliminated the need for any FIB milling beyond the initial step to fabricate the imprint mold. In this paper, we provide experimental details on the imprint of campanile probes and present actual NSOM measurements demonstrating that fully functional nano-optical probes can be fabricated using NIL.

## Materials and Methods

To determine the optimal campanile structure, 3D finite-difference time domain (FDTD) simulations were performed using a commercial software suite (Lumerical). The simulated structure reported here provides an optimal balance between high optical performance and robustness during fabrication. Light with a fiber mode field distribution was injected from the base of the campanile structure. The mode field diameter was chosen to be 4.2 µm, which corresponds to a commonly used S630-HP single mode fiber. The optical constants of gold and the dielectrics of the fiber core were taken from Johnson and Christy^30^, and the manufacturer’s specification, respectively^31^. The field strength was measured at 5 nm above the apex of the tip, and the field enhancement was calculated as $\frac{|{E}_{{\max }}|}{|{E}_{0}|}$, where $|{E}_{{\max }}|$ is the maximum field amplitude in the measurement plane and $|{E}_{0}|$ is the maximum field amplitude in the incoming fiber mode. The gap at the tip apex was chosen to be 20 nm and the mesh size in the region near the tip is 2.5 nm. The mesh size is nonuniform and increases gradually up to ~25 nm in the uniform dielectric regions. Perfectly matched layers were used for all the boundaries. Figure 1 depicts the simulation results that compare a conventional campanile tip with a towered base produced by conventional FIB milling (Fig. 1a,c)^21, 22^ and the simplified structure without the tower (Fig. 1b,d). While the simulations show that the presence of tower can slightly increase the field enhancement, the difference is small. Therefore, to simplify the fabrication we chose to fabricate a campanile probe consisting of only a large pyramidal structure. The movie files showing the electric field distribution as a function of time can be found in the supporting information.

**Figure 2:**
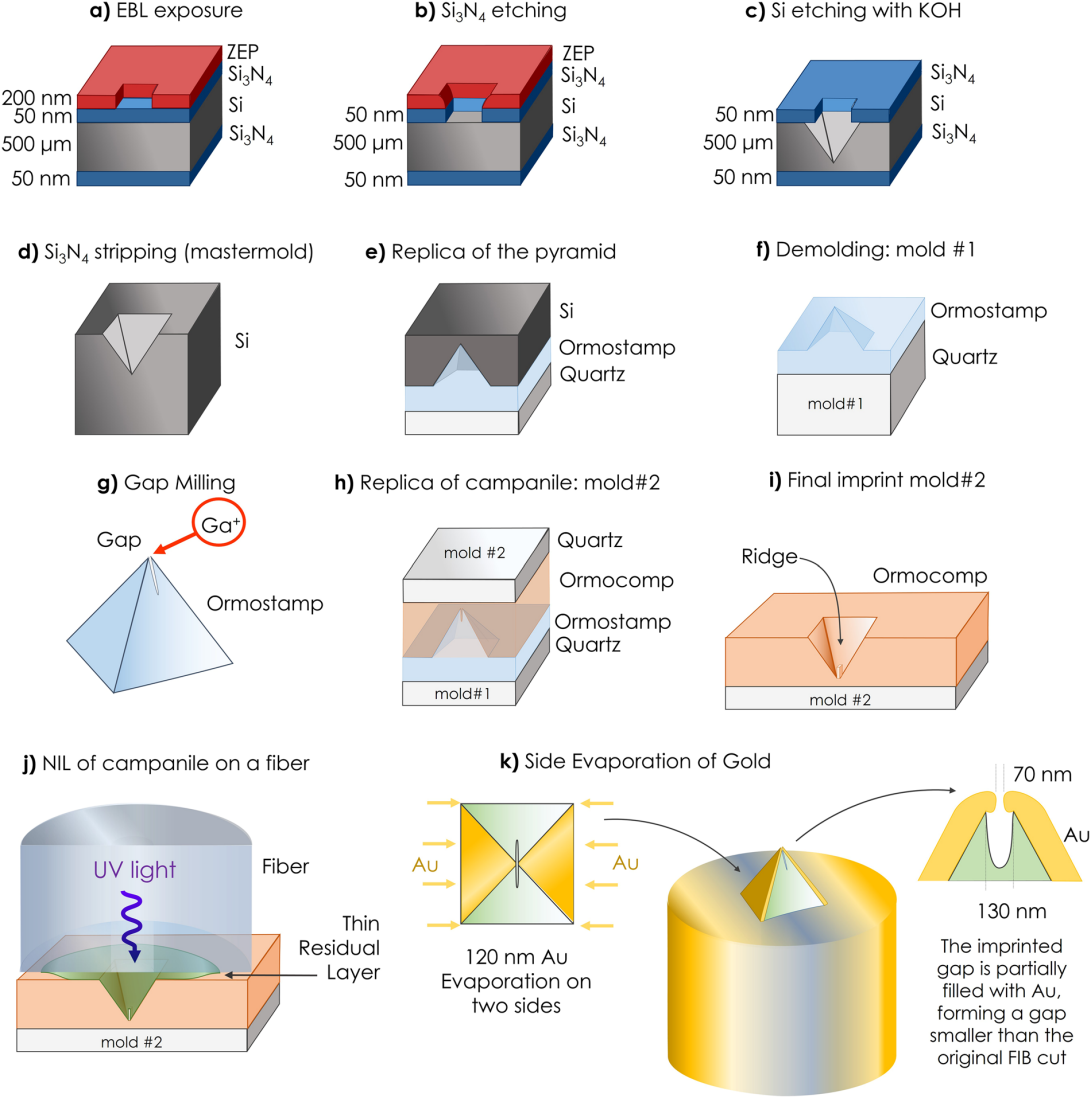
Fabrication process. **(a)** EBL exposure and development of ZEP. (**b)** Pattern transfer into Si_3_N_4_ by RIE. (**c)** The undercut of silicon in KOH to form inverted pyramids. (**d)** Si_3_N_4_ stripping and completion of the mastermold. (**e**) Replication of the mastermold into Ormostamp to form pyramids. (**f**) Demolding and completion of mold #1. (**g**) Ga+ FIB milling of the gap at the apex of the pyramid. (**h**) Replication of milled pyramids into Ormocomp. (**i**) Demolding and completion of mold #2 on a quartz substrate. (**j**) Imprint on a fiber using the process described in^28^. (**k**) Evaporation of 120 nm Au on two of the four sides of the pyramid. The imprinted slit at the apex prevents gold from shortcutting the two sides and creates a plasmonic gap at the tip of the probe.

The fabrication strategy adopted here includes imprinting a pyramid on a fiber using a transparent, imprintable resin that will function as the dielectric part of the MIM structure. The imprinted pyramid is then sandwiched by metal, whereby two of the four sides of the pyramid are evaporated with gold. The complete fabrication workflow is reported in Fig. 2 and starts with the fabrication of the imprint mastermold for the pyramid. The mastermold substrate consists of a double-polished silicon wafer (100) coated with 50 nm of Si_3_N_4_ on both sides. An electron beam resist (ZEP520A) is spincast on one side of the wafer until a thickness of about 60 nm is reached. Square patterns with a base size of 5 µm are exposed to ZEP using a Vistec VB300 EBL system (Fig. 2a). Development the ZEP is performed in amyl acetate, and pattern transfer into silicon nitride is performed by reactive ion etching (RIE) using a mixture of CHF_3_ and O_2_ (Fig. 2b). Inverted pyramids are obtained by etching the silicon in a KOH bath (Fig. 2c) and then stripping the Si_3_N_4_ in a bath of phosphoric acid (Fig. 2d). Replication of the mastermold in Ormostamp^31^ enables inversion of the tonality of the lithography and results in mold #1 (Fig. 2e and f). A slit of about 130 nm in width is milled at the apex of the pyramid in mold #1 using the 30 keV gallium FIB of a Zeiss ORION NanoFab microscope (Fig. 2g). The purpose of the slit is ultimately to prevent the evaporated gold films from the opposing faces of the pyramid from contacting and forming a short, which destroys the near-field performance of the tip. Mold #1 is replicated on a transparent substrate using Ormocomp^31^, yielding mold #2 (Fig. 2h and i). Mold #2 is used to imprint the gapped-pyramid directly onto the facet of an optical fiber (step 2i to 2j) using a custom-made setup with a co-axial alignment capability. The process has been previously established and the full fabrication details can be found in ref. 28.

The precise coaxial alignment between the fiber core and pyramid is performed with an inverted microscope using a piezo-stage. The process is guided by a red laser light coupled into the opposite end of the optical fiber. Ormocomp^31^ (RI = 1.52) is used as the imprint resin and waveguiding medium. Upon contact, the optical fiber is pushed against mold #2 to have an optimal filling of the pyramid and minimize the residual layer between the face of the fiber and the base of the pyramid. After demolding, 120 nm of gold is evaporated on two sides of the pyramid (Fig. 2k). Because of the combined effects of the geometry of the thick gold films and the direction of the evaporation, the resulting metal gap becomes smaller than the FIB-milled slit (mold #1).

## Result and Discussion

**Figure 3:**
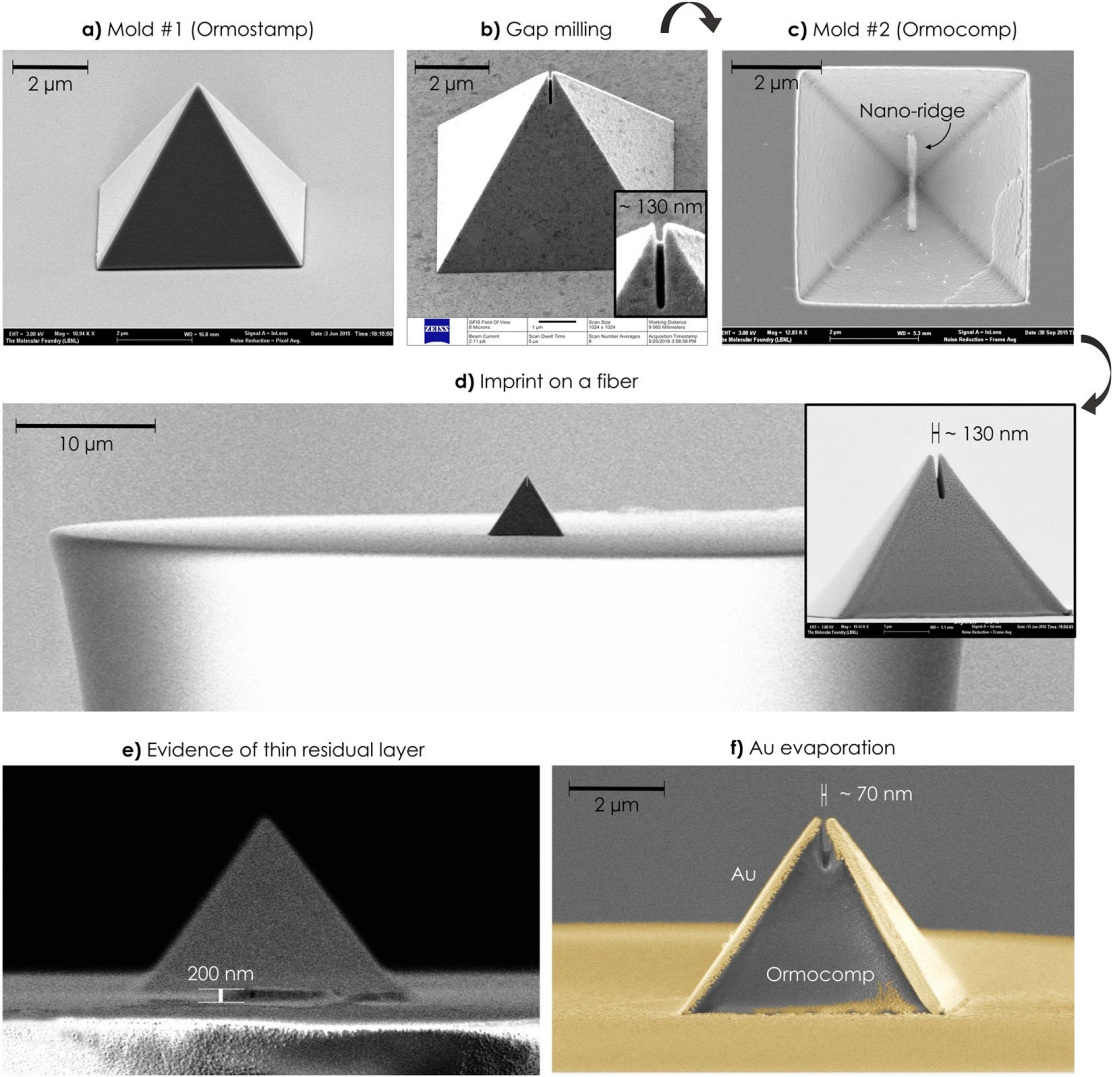
Micrograph of the fabrication steps. (**a**) Tilted SEM view of mold #1 (~75°) showing polymeric pyramid on a hard substrate replicated from the silicon mastermold. (**b**) Gap milling on mold #1 using Ga^+^-beam (tilted HIM view in Zeiss ORION NanoFab microscope, 54°). (**c**) SEM top view of mold #2, which is a replica of mold #1 in Ormocomp on a transparent support to allow imprint on the fiber. (**d**) Tilted-view (~90°) SEM of the final pyramid imprinted on an optical fiber. The inset shows a close-up of the campanile on the fiber that has a gap of about 130 nm in width at the apex. A negligible amount of superficial roughness is visible and is caused by sputtered Au-Pd added for imaging purposes. (**e**) Same view as d (but rotated 90°) that shows a residual layer of about 200 nm. (**f**) False-color SEM image of the completed campanile probe after side-evaporation of Au. The evaporated metal reduces the width of the imprinted slit, yielding a gap of about 70 nm.

Figure 3 presents a collection of scanning electron microscopy (SEM) and helium ion microscopy (HIM) micrographs of the main fabrication steps. Figure 3a is a tilted-view image of a pyramid from mold #1. Ormostamp’s low viscosity and advantageous filling properties ensure that imprinted pyramids have a very sharp tip. Thanks to the focused nature of the Ga-beam, minimal rounding of the soft polymeric tip is observed after the Ga-FIB milling step of the gap at the apex (Fig. 3b). Figure 3c is a top-view image of mold #2 which is used as the final imprint mold to replicate the campanile probe onto the facet of the fiber that has a diameter of about 65 µm (Fig. 3d). The inset in Fig. 3d demonstrates the remarkable accuracy of this lithography process, which retains sharp features and high-resolution patterns – e.g. the gap at the tip of the probe – despite the challenging imprint on the small surface area of a fiber facet. Furthermore, Fig. 3e proves that a residual layer less than 200 nm in thickness can be achieved by proper design of the imprint process^28^. Figure 3f is a false color SEM image of the imprinted probe that has undergone evaporation of gold on two opposite sides of the pyramid. As mentioned above, the imprinted slit prevents shorting of the metalized sides and forms a gap of about 70 nm, which ultimately determined the near-field imaging resolution of the probe as discussed below.

**Figure 4:**
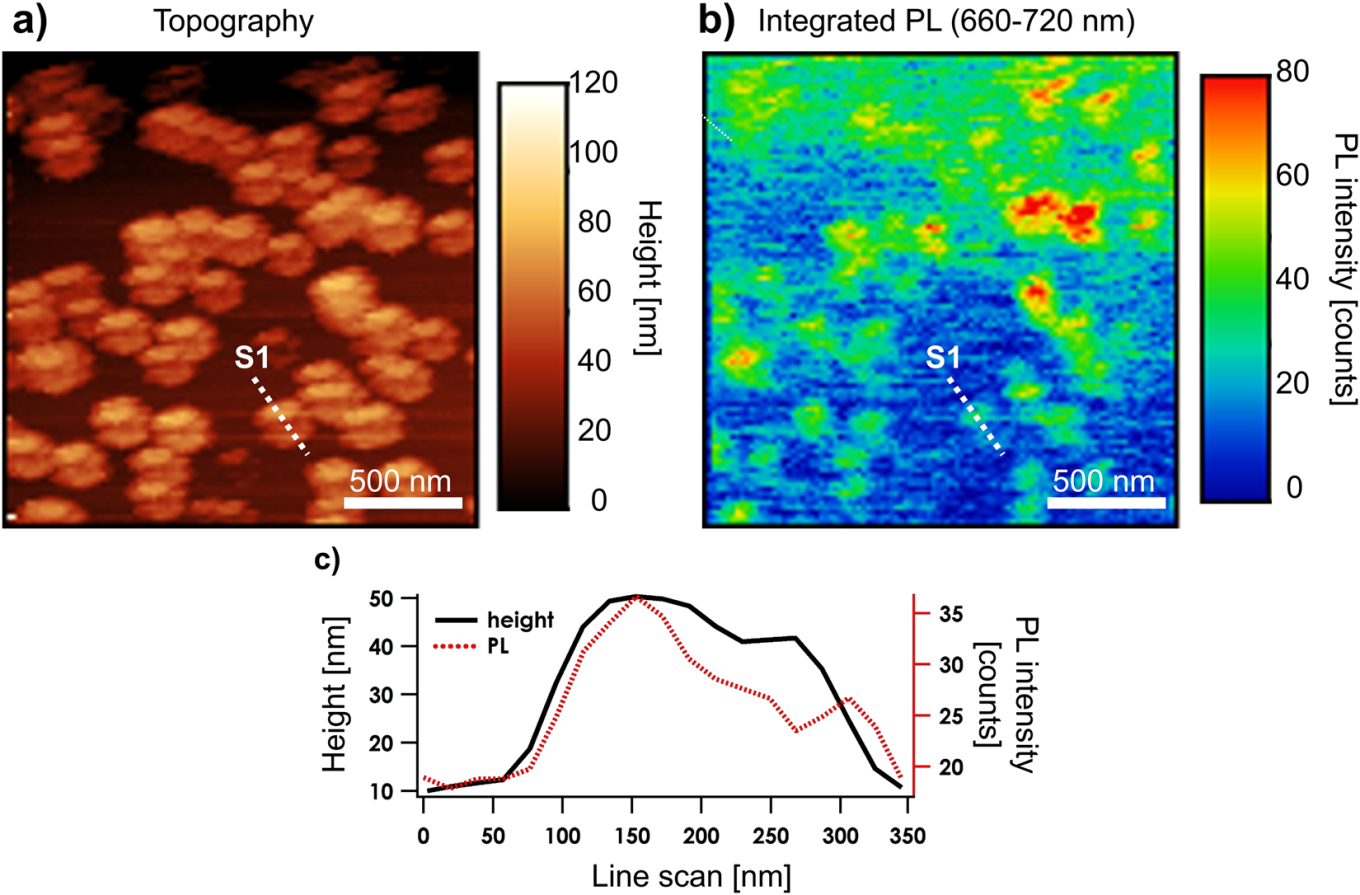
Near-field scan results. Correlated height map (**a**) and integrated PL map (2 × 2 µm^2^) (**b**) of isolated 40 nm diameter fluorescent beads obtained using an imprinted campanile probe. (**c**) Line scans from the topography and PL maps.

The functionality of the imprinted probes was tested using a scanning near-field optical microscope in a shear-force configuration (NTEGRA Spectra, NTMDT) as described in ref. 22. Standard atomic force microscopy (AFM) feedback algorithms are employed to maintain a constant phase delay, which maintains a tip-sample distance of ~5 nm while the sample is scanned laterally with precision xy piezo stages. Optical excitation is achieved by coupling ~50 µW of a 633 nm HeNe continuous wave laser into the optical fiber with the imprinted campanile probe. Calibration measurements were performed on a dispersed film of standard 40 nm polystyrene beads containing fluorescent dyes (AlexaFluor 680) with excitation and emission maxima wavelengths of 633 nm and 680 nm, respectively. While excitation wavelength lies at the edge of campanile efficiency, the collection wavelength of ~700 nm lies well within the working range of the campanile probes. Nano-photoluminescence (nano-PL) was excited and collected using the imprinted campanile probe, providing nearly background-free imaging. Figure 4a shows a 2 × 2 um^2^ topography scan of the polystyrene beads, which exhibits a “double image” feature (due to the metal-gap-metal nature of the tips) that is uniformly oriented for all of the beads. This feature is commonly observed with traditional FIB-fabricated campanile probes and is a hallmark of a functioning optical probe since it shows that the campanile structure has a gap feature at the apex of the tip. Most importantly, we see that the PL emission from the beads can be mapped with the imprinted probes, appearing as ~80 nm spheres as shown in Fig. 4b and c. Note that the gradient in intensity from the top to the bottom of Fig. 4b is due to a slow drift in the optical coupler that couples the excitation laser into the tip. Nevertheless, the calibration scans clearly demonstrate that the imprinted campanile tips enable sub-diffraction-limited imaging with a resolution determined by the gap size, thus demonstrating their functionality as well as the promise for (much) higher throughput campanile tip fabrication using the NIL method. Additional measurement results are provided in the Supporting Information to demonstrate the near-field proficiency including polarization and distance dependence of the photoluminescence signal.

## Conclusion

In this work, we report a simple NIL-enabled fabrication of a 3D optical transformer directly onto the fiber. We demonstrate the capability to imprint 3D structures with sub-70 nm scale features and sub-100 nm positioning precision, creating functional ‘campanile’ near-field probes. The functionality of the imprinted probes was validated by performing hyperspectral nano-PL mapping of a dispersed film of 40 nm polystyrene fluorescent beads. Both the topography scans and the PL maps exhibited features consistent with the behavior of traditional FIB-fabricated optical transformer probes. Results show that imprinted campanile probes enable sub-diffraction-limit imaging with a resolution of ~ 80 nm, determined by the gap size at the tip apex. The fabrication process presented here can be scaled and parallelized for higher throughput and offers a promising route for mass production of nano-optical devices.

## Electronic supplementary material

Supplementary Information

Field Profile Campanile with Tower

Field Profile Campanile without Tower
