# Supplementary material for: Campanile Near-Field Probes Fabricated by Nanoimprint Lithography on the Facet of an Optical Fiber
Source: Sci Rep. 2017 May 10;7:1651. doi: 10.1038/s41598-017-01871-5 (PMC5431761; doi:10.1038/s41598-017-01871-5)
Supplement: Supplementary file 1 — Supplementary Information [file 41598_2017_1871_MOESM1_ESM.pdf]

Supporting Information

Campanile Near-Field Probes Fabricated by Nanoimprint Lithography on the Facet of an Optical Fiber

Giuseppe Calafiore[1*], Alexander Koshelev[1*], Thomas P. Darlington[2*], Nicholas J. Borys[2], Mauro Melli[2], Aleksandr Polyakov[2], Giuseppe Cantarella[2], Frances I. Allen[2,3,4], Paul Lum[3], Ed Wong[2], Simone Sassolini[2], Alexander Weber-Bargioni[2], P. James Schuck[2], Stefano Cabrini[2], Keiko Munechika[1†]

[1] *aBeam Technologies, Hayward, CA 94541, USA*

[2] *The Molecular Foundry, Lawrence Berkeley National Laboratory, One Cyclotron Road, Berkeley, CA 94720, USA*

[3]*Biomolecular Nanotechnology Center/QB3, Stanley Hall, University of California, Berkeley, CA 94720, USA*

[4]*Department of Materials Science and Engineering, University of California, Berkeley, CA 946720, USA*

[†]Corresponding author: km@abeamtech.com

* A. Koshelev, G. Calafiore, and T. Darlington contributed equally to this work. Correspondence should be addressed to K.M. (email: km@abeamtech.com).

Supporting Information 1: Videos of electric field distribution vs. time inside the metal-insulator-metal (‘campanile’) geometry:

The videos show electric field distribution as a function of time inside campanile with (a) and without (b) tower. The distribution is presented in the logarithmic scale identical to the one used in the main Figure 1.

Field-profile-campanile-with-tower(1).avi

Figure S1-(a) : Electric field distribution for a ‘campanile’ with a tower

Field-profile-campanile-without-tower(1).avi

Figure S1-(b) Electric field distribution for a ‘campanile’ without a tower

Supporting Information 2:

Figure SI-1 shows the FDTD simulation results with the experimental gap width of 70 nm.

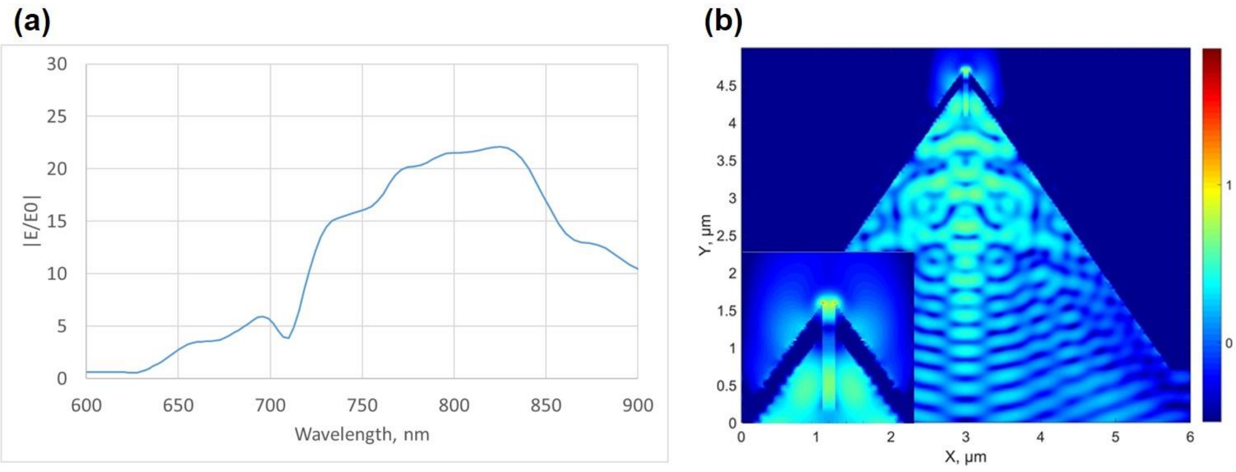

Figure SI-1: Field enhancement of the “tower-less” campanile with a 70 nm gap. (b) FDTD simulations of the electric field amplitude inside the campanile with a 70 nm gap.

Supporting Information 3:

Figure SI-2 shows nano-PL characterization of a flake of monolayer WSe_2 – a luminescent, atomically thin 2D semiconductor – using a campanile near-field probe produced with NIL. Figure SI-2(a) shows the nano-PL map of the WSe_2 flake. The dashed line indicates the line cut across the edge of the flake which was used for the intensity profile that is plotted in Figure SI-2(b). Across the edge, the PL is found to diminish from a its maximum to minimum value over ~150 nm which sets an upper limit to the spatial resolution of the tip. The nano-PL intensity did not show any correlation with topographic features of the WSe_2. This lack of correlation is expected given the relatively flat, 2D nature of monolayer WSe_2.

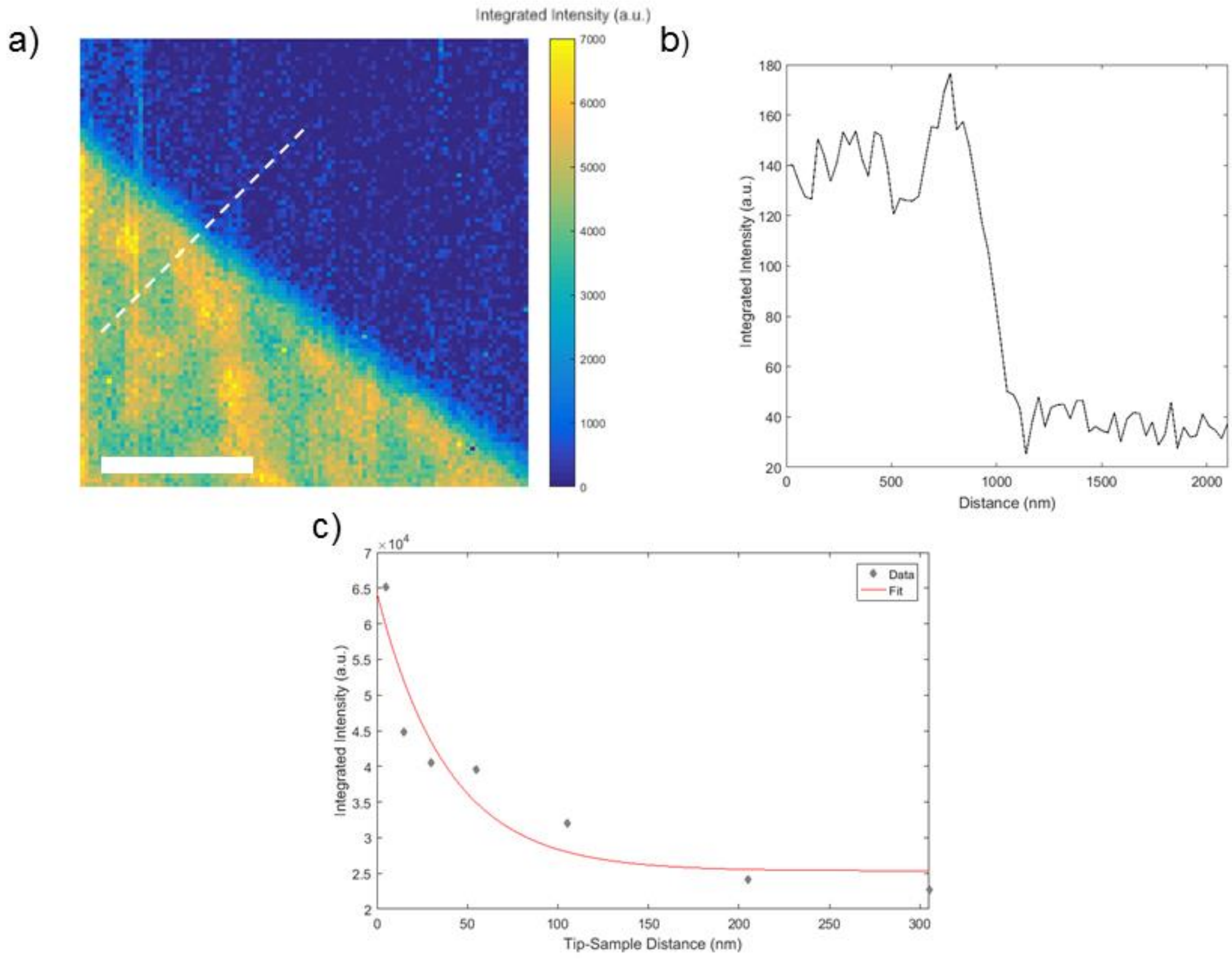

Figure SI-2: Characterization of a campanile near-field probe produced by NIL. (a) Nano-PL intensity map of the edge of a flake of monolayer WSe_2; scale bar: 1 µm. (b) Intensity profile of the dashed line in panel (a). (c) Integrated PL intensity plotted as a function of distance of the tip from the sample surface. The decay of the near-field signal shows an exponential decrease with a characteristic decay length of ~40 nm (fit shown as the red line).

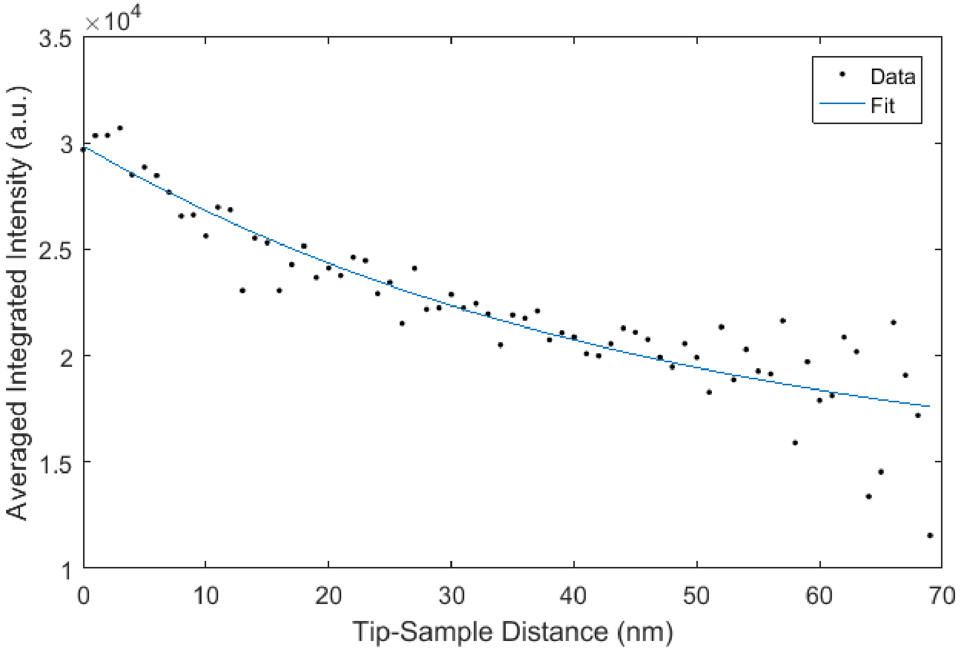

Figure SI-3: Spacer-based statistical characterization of a campanile near-field probe produced by NIL. A transparent dielectric spacer with spatially varying thickness was used to measure the average dependence of the integrated intensity of the nano-PL from monolayer WSe_2 over tip-sample separations ranging from 0 – 70 nm. Similar to the results shown in Fig. SI-2, we find an approximately exponential dependence of the nano-PL signal with a characteristic decay length of ~47 nm (fit shown as the blue line).

SNOM Measurement Procedure:

The scanning probe system used was a NT-MDT NTEGRA Spectra scanning near-field optical microscope with a customized shear configuration. The imprinted aBeam campanile tip was first attached to 150 kHz quartz tuning fork with a resonance Q-factor after gluing of ~500. Excitation was performed using a Melles Griot He-Ne 632.8 nm laser source, with a total power of ~70 uW coupled into the SM optical fiber of the tip. Polarization was controlled via a lambda half-wave plate prior to coupling into the optical fiber of imprinted campanile tip. Hyperspectral nano-PL map was detected with a cooled CCD camera (Andor iDus CCD DV401A-BV-600), with a 1 sec integration time.

For all nano-PL measurements a background spectra was subtracted to remove Raman signal due to the silica core of the SM optical fiber. This background changes over time due to thermal drift of the coupling efficiency of the laser in the optical fiber. To account for this, an adaptive scaling method was used to normalize the silica Raman backgrounds of each individual spectra in Fig. SI-2 (a). An analogous procedure was used for the individual spectra Fig. SI-2 (b). All optical data was processed using Mathworks MATLAB Computational Suite (Version R 2016b).
